# Association of dual-task walking performance and leg muscle quality in healthy children

**DOI:** 10.1186/s12887-015-0317-8

**Published:** 2015-02-05

**Authors:** Rainer Beurskens, Thomas Muehlbauer, Urs Granacher

**Affiliations:** Department of Health and Sports Sciences, Division of Training and Movement Sciences, Research Focus Cognition Sciences, University of Potsdam, Am Neuen Palais 10, Bldg. 12, D-14469 Potsdam, Germany

**Keywords:** Gait, Cognitive interference, Body composition, Muscle mass, Children

## Abstract

**Background:**

Previous literature mainly introduced cognitive functions to explain performance decrements in dual-task walking, i.e., changes in dual-task locomotion are attributed to limited cognitive information processing capacities. In this study, we enlarge existing literature and investigate whether leg muscular capacity plays an additional role in children’s dual-task walking performance.

**Methods:**

To this end, we had prepubescent children (mean age: 8.7 ± 0.5 years, age range: 7–9 years) walk in single task (ST) and while concurrently conducting an arithmetic subtraction task (DT). Additionally, leg lean tissue mass was assessed.

**Results:**

Findings show that both, boys and girls, significantly decrease their gait velocity (*f = 0.73*), stride length (*f = 0.62*) and cadence (*f = 0.68*) and increase the variability thereof (*f = 0.20-0.63*) during DT compared to ST. Furthermore, stepwise regressions indicate that leg lean tissue mass is closely associated with step time and the variability thereof during DT (*R*^2^ = 0.44, p = 0.009). These associations between gait measures and leg lean tissue mass could not be observed for ST (*R*^2^ = 0.17, p = 0.19).

**Conclusion:**

We were able to show a potential link between leg muscular capacities and DT walking performance in children. We interpret these findings as evidence that higher leg muscle mass in children may mitigate the impact of a cognitive interference task on DT walking performance by inducing enhanced gait stability.

## Background

Epidemiologic studies indicate that the risk of sustaining a fall is particularly high in children and seniors [1,2] and a large number of falls occur during ambulation [3]. The control of human walking has traditionally been considered an automatic process that only requires minimal cognitive effort. However, recent research using dual-task (DT) paradigms showed evidence that the control of locomotion requires cognitive resources (cf. [4] for a review). Only few studies explored the ability of children to perform a cognitive and a walking task simultaneously. Dual-task walking in children causes, among others, a reduction in gait speed and stride length and an increase in step time and double-limb support time [5,6]. Their motor abilities are most likely restricted by maturational deficits [7].

The reasons for impaired balance performance in children have been attributed to not fully developed structures within the central nervous system [8]. For example, Riach and Hayes [8] investigated age-related changes in postural sway in children and compared their findings to results from adult research. They were able to show that children predominately rely on visual information to control balance, whereas grown-ups prioritize the proprioceptive system. In this context, Peterson et al. [9] observed that children at the age of 12 years develop adult-like abilities to integrate proprioceptive feedback in balance control. Children often encounter situations involving the concurrent performance of a cognitive task while walking. For example, they may need to identify signs and signals on their way to school or talk to classmates and carry a book or physical education utilities while walking. Children aged 9 years show impaired motor performance when walking in DT situations compared to young adults [10]. Especially, young children (4–6 years) decrease their stride length and increase the variability of temporal and spatial gait parameters when walking in a motor-demanding DT situation (e.g., carrying a box) [6]. A similar interference can be seen during walking while concurrently performing attentional-demanding cognitive tasks [6,11,12]. It has been reported that children develop a slower gait, take shorter steps, and increase their stride time during walking while performing Stroop-like tasks [11], non-verbal memory tasks [12], or arithmetic tasks [6]. These findings indicate that children tend to change their gait behavior during dual-tasking to adopt a more cautious gait pattern [13]. The mentioned declines in the primary (postural task) and/or the secondary task (cognitive or motor interference task) have been explained by limited cognitive capacities [14] or cognitive interferences when two tasks share cognitive/sensory modalities and processing resources [15].

Besides the aforementioned cognitive capacity [4], walking performance, especially in the elderly, is additionally affected by leg muscle weakness [7] and deteriorated postural control [16]. Moreover, it has been reported that children’s neuromuscular system and cognitive functioning is impaired due to maturational deficits [10,17]. An approach that received little attention is the relationship between body composition (e.g., muscle mass) and motor functions. To our knowledge, there is no study available that investigated the relation between lower extremity muscular capacity and walking in children. This is surprising because muscular capacity in children is associated with physical activity [18], indicating that physically active children are less obese and have higher amounts of muscle mass. In fact, children who have low levels of body fat and mass tend to perform better on physical fitness tests and develop improved motor coordination [19], which might affect their performance during DT walking. Improved coordinative skills in children may lead to less cognitive control needed to control movements, which might free up cognitive resources needed to concurrently perform a primary walking task and a secondary cognitive task [7]. However, it still remains open to what extent the muscular capacity of prepubescent children is related to their DT motor performance, i.e. their ability to concurrently walk and perform a cognitive interference task.

Thus, the purpose of the present study was to investigate the influence of a concurrent arithmetic cognitive task on locomotion in prepubescent children and to examine associations thereof with measures of leg muscle capacity. An age range of 7–9 years was chosen to insure that the children are old enough to follow the study protocol but young enough to demonstrate interference effects distinct from those of adults [12]. We hypothesize that a) spatio-temporal gait parameters (e.g., gait velocity, stride time) will decrease during DT compared to single-task (ST) walking and the variability thereof will increase and (b) changes in DT motor control are associated with measures of body composition (i.e., leg muscle quality).

## Methods

### Participants

A group of 20 prepubescent children participated in this study; their characteristics are summarized in Table 1. Pubertal status was self-reported by the participants of the study and pubic hair development was reported for girls and for boys. Classification of pubertal status was done according to Marshall and Tanner [20]. Children had no known neuromuscular diseases or attentional deficits according to parent’s reports and none of them had participated in research on gait or cognition within the preceding 6 months. Subject’s physical activity was assessed using a self-report questionnaire that included overall physical activity during a normal week, everyday physical activity (duration, frequency, type), sports activity at school as well as in and outside organized clubs (duration, frequency, intensity, type, seasonality) [21]. The Human Ethics Committee at the University of Potsdam approved the study protocol (reference number: 25/2014). Before the start of the study, each participant and their parents/guardians read, concurred, and signed a written informed consent. All procedures were conducted according to the Declaration of Helsinki. An a priori power analyses using 2 groups and a repeated measure ANOVA design yielded a total sample size of *N* = 18 (effect size [*f*] = 0.4, α = 0.05), with an actual power of 0.88 (critical *F*-value = 4.49).

**Table 1:** **Characteristics of the study participants**

**Characteristic**	**Total (n = 20)**	**Male (** ***n*** **= 10)**	**Female (** ***n*** **= 10)**
Age [years]	8.6 ± 0.7	8.8 ± 0.8	8.3 ± 0.5
Height [cm]	139.9 ± 6.3	141.5 ± 6.6	138.3 ± 5.7
Mass [kg]	32.4 ± 4.9	31.6 ± 2.4	33.1 ± 6.7
BMI [kg/m^2^]	16.7 ± 2.4	15.9 ± 1.5	17.5 ± 2.9
Tanner stage^1^	1.2 ± 0.4	1.0 ± 0.0	1.4 ± 0.5
Physical activity level [h/wk]	7.4 ± 3.9	6.8 ± 3.3	8.0 ± 4.7
LTM-LE (kg)	3.7 ± 0.7	3.9 ± 0.7	3.4 ± 0.7

Note: ^1^Pubic hair development was self-reported by the participants. BMI = body mass index, LTM-LE = lean tissue mass of the lower extremities.

### Experimental procedures

The experiment was subdivided into 2 walking conditions. Participants walked with their own footwear at self-selected, comfortable walking speeds, initiating and terminating each walk a minimum of 2 m before and after a 10-m walkway to allow sufficient distance to accelerate and decelerate from a steady-state of ambulation across the walkway. One recorded trial led to the registration of 13–18 steps (i.e., 6–9 strides), which has been shown to be sufficient to analyze walking behavior. In fact, Besser and colleagues [22] reported that 5–8 strides are necessary for 90% of the individuals to obtain reliable mean estimates of spatio-temporal gait parameters. During ST condition, participants were asked to walk along the straight pathway of 10 m length. In DT condition, participants walked along the pathway while performing a concurrent attention-demanding cognitive interference task. The interference task was an arithmetic task, where participants were instructed to recite out loud serial subtractions by 3 starting from 100. Both tasks were performed in a counterbalanced order and each walking condition included one familiarization trial ahead of the test trial. The latter trial was used to collect the behavioral data included in our statistical analyses.

### Gait analyses

Participant’s walking performance was registered using a 10-m instrumented walkway equipped with an OptoGait-System (Microgait, Bolzano, Italy). The OptoGait-System is an opto-electrical measurement system consisting of light-transmitting and -receiving bars. Each bar is 1 m in length and is composed of 100 LEDs that continuously transmit to an oppositely positioned bar. With a continuous connection between two bars, any break in the connection can be measured and timed. The walking pattern was registered at 1 kHz, allowing the collection of spatial and temporal gait data. The OptoGait-System demonstrated high discriminant and concurrent validity with a validated electronic walkway (GAITRite®-System) for the assessment of spatio-temporal gait parameters in healthy subjects [23]*.* We defined *gait velocity* as distance in meter covered per second during 1 stride, *stride length* as the linear distance (cm) between successive heel contacts of the same foot. Additionally, *stride time* was defined as the time (s) between the first contacts of 2 consecutive footfalls of the same foot and *cadence* as estimated number of strides per minute. We then calculated mean and standard deviation (SD) of each gait measure. In addition, coefficients of variation (CV) for gait velocity, stride length, and stride time were calculated according to the formula: $CV=\left(\frac{SD}{Mean}\right)\times 100$.

### Assessment of body composition

Participant’s body composition was assessed using non-invasive bioelectrical impedance analysis (BIA). An octopolar tactile-electrode impedance meter (InBody 720, BioSpace, Seoul, Korea) was used to estimate body composition. The InBody 720-System uses 8 electrodes (i.e., 2 in contact with the palm and thumb of each hand, 2 with the anterior and posterior aspects of the sole of each foot) and applies alternating currents of 250 mA at frequencies of 1, 5, 50, 250, 500, and 1,000 Hz to detect resistance of the different body segments. During testing, subjects stood in upright quiet stance with bare feet on a footplate and held electrodes in both hands. Whole-body resistance was then calculated as the sum of each segmental resistance (i.e., right arm, left arm, trunk, right leg, left leg). BIA using the InBody 720-System has been validated by dual-energy X-ray absorptiometry (R^2^ = 0.93) [24]. For statistical analyses, we included the lean tissue mass of subject’s lower extremities (LTM-LE as the mean of the left and right leg). LTM-LE of BIA measured with InBody 720-System is highly correlated with leg skeletal muscle mass (SMM) measured with DEXA (R^2^ = 0.79) [25].

### Statistical analyses

Data are presented as group mean values ± standard deviations. To assess overall condition-related effects on walking performance, a one-way analyses of variances (ANOVA) with the within-factor Condition (ST vs. DT) was computed. To investigate sex-differences, a 2 (sex: female, male) x 2 (condition: ST, DT) ANOVA with Condition as repeated within-subject factor was used to analyze walking performance. The classification of effect sizes (*f*) was determined by calculating partial eta-squared (*eta*^2^). The effect size is a measure that describes the effectiveness of a treatment and it helps to determine whether a statistically significant difference is a difference of practical concern. Effect sizes can be classified as small (0.00 ≤ *f* ≤ 0.24), medium (0.25 ≤ *f* ≤ 0.39), and large (*f* ≥ 0.40). Correlation analyses and stepwise linear regression analyses were used to asses associations between LTM-LE and walking measures. Correlations are reported by their correlation coefficient *r* and their Bonferroni-corrected *p*-value; associations are reported by their coefficient of determination (*R*^*2*^) and the corresponding level of significance. Variables were added stepwise, with the inclusion and exclusion criterion of *p* < 0.05. All analyses were calculated using Statistical Package for Social Sciences (SPSS) version 22.0 (IBM Corp., New York, USA) and significance levels were set at α = 5%.

## Results

**Figure 1:**
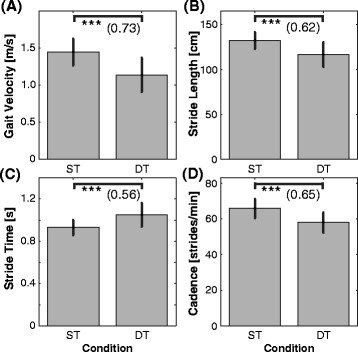
**Means and standard deviations for each gait measure** (**A** **: gait velocity,** **B** **: stride length,** **C** **: stride time,** **D** **: cadence) and each walking condition separately.** Asterisks show significance levels (***, **, *, n.s. represents *p* < 0.001, *p* < 0.01, *p* < 0.05, and non-significant [*p* > 0.05], respectively); Effect size (*f*) is displayed in brackets. ST = single-task walking; DT = dual-task walking.

**Figure 2:**
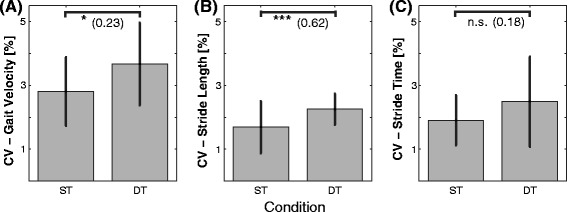
**Coefficient of variation (CV) for three stride-related gait measures (A: CV - gait velocity, B: CV - stride length, C: CV - stride time) and each walking condition separately.** Asterisks show significance levels (***, **, *, n.s. represents *p* < 0.001, *p* < 0.01, *p* < 0.05, and non-significant [*p* > 0.05], respectively). Effect size (*f*) is displayed in brackets. ST = single-task walking; DT = dual-task walking.

Figure 1A-D display means and SDs of our 4 measures of walking performance and Figure 2A-C show the respective CV measures for gait velocity, stride length, and stride time; separately for each walking condition. The corresponding ANOVA outcomes are displayed in Table 2.

**Table 2:** **ANOVA outcome**

	**Means ± SD**		**p-value (f)**
	**ST**	**DT**	
gait velocity [m/s]	1.45 ± 0.2	1.14 ± 0.2	< 0.001 (0.73)
stride length [cm]	132.53 ± 2.1	116.57 ± 13.6	< 0.001 (0.62)
stride time [s]	0.93 ± 0.1	1.05 ± 0.1	< 0.001 (0.57)
cadence [strides/min]	65.79 ± 5.3	57.99 ± 5.7	< 0.001 (0.68)
CV - gait velocity [%]	5.62 ± 2.2	7.34 ± 2.6	0.03 (0.24)
CV - stride length [%]	3.38 ± 1.6	4.51 ± 0.9	< 0.001 (0.63)
CV - stride time [%]	3.81 ± 1.6	5.50 ± 3.6	0.06 (0.20)

Note: CV = coefficient of variation; *f* = effect size; ST = single-task walking; DT = dual-task walking; n.s. = non-significant. Subdividing subjects according to their sex (male/female) and including this factor in the ANOVA did not show any sex-related significance (all *p* > 0.05).

The results show that participants walked significantly slower (22%, *f* = 0.73), took shorter steps (12%, *f* = 0.62), increased their stride time (13%, *f* = 0.56), and decreased their cadence (12%, *f* = 0.73) during DT compared to ST walking (Figure 1A-D). With reference to measures of gait variability, participants showed significantly increased spatio-temporal variability in 2 out of 3 measures during DT walking (i.e., CV - gait velocity: *f* = 0.24, CV - stride length: *f* = 0.63; cf. Figure 2A-C). To ensure that the observed changes in gait variability are not linked to the reduction in mean gait velocity, we added gait velocity as a covariate into our analyses of co-variances (ANCoVA). Gait velocity did not significantly affect coefficients of variation in gait velocity (*p* = 0.08), in stride length (*p* = 0.82), and in stride time (*p* = 0.89), indicating that the investigated changes in gait variability are independent from the reduction in gait velocity during DT walking. The inclusion of the factor “sex” in our ANOVA model did not change our findings (all *p* > 0.05).

**Figure 3:**
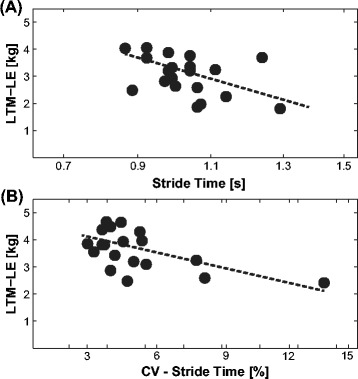
**Correlations of subject’s leg lean tissue mass** **with stride time** **(A)** **and CV of stride time** **(B).** Regression analysis yielded significant associations between stride time, the CV thereof, and subject’s LTM-LE (*R* ^2^ = 0.44; *p* = 0.009) during dual-task walking.

Pearson’s correlation analyses with Bonferroni-corrected *p*-values of LTM-LE and measures of gait indicated non-significant, small sized correlations, irrespective of the measure considered. Furthermore, LTM-LE was not significantly correlated with age (*r* = 0.37; *p* = 0.1). Of note, we observed an unequivocal tendency indicating that participants with less LTM-LE walked slower (*r* = 0.41; *p* = 0.42) and took shorter steps (*r* = −0.43; *p* = 0.35) with larger variability of gait velocity (*r* = −0.38; *p* = 0.54), and stride time (*r* = −0.56; *p* = 0.07) during DT walking. To further estimate associations between subject’s gait measures and LTM-LE, we performed stepwise linear regression analyses. During ST, regression did not show significant associations (*R*^2^ = 0.17; *p* = 0.19). In contrast, during DT, regression analysis yielded a significant association between stride time, the CV thereof, and subject’s LTM-LE (*R*^2^ = 0.44; *p* = 0.009; Figure 3A-B).

## Discussion

The present study was designed to describe the gait behavior of prepubescent children aged 7–9 years while walking in a cognitively challenging DT situation. We examined the effects of a concurrent secondary task on children’s locomotor system and its relationship with correlates of lower extremity muscle mass. To this end, we combined walking with an arithmetic task (i.e., serial subtractions by 3), a task that proved to decrease locomotor performance in young and older adults [26]. In general, the results showed that normal walking was affected when children had to perform a concurrent secondary task, irrespective of their sex. Gait velocity, stride length and cadence decreased and stride time as well as spatio-temporal variability measures (i.e., CV in gait velocity and stride length) increased in boys and girls during DT walking. Furthermore, significant associations were found between children’s leg muscular capacity and DT walking performance.

These findings are consistent with previous studies investigating DT performance in children [5,6]. Further, similar results were found for older adults during DT walking [10], indicating that DT performance decreases in seniors and children. In general, the magnitude of decrease in gait velocity in our study resembles the changes found in previous studies [5], where children decreased their gait velocity by 0.18 and 0.43 m/s, depending on the secondary task used (i.e., memorization task and auditory identification task, respectively). In the present study, children significantly reduced their gait velocity by 0.31 m/s and increased the variability thereof, indicating that the cognitive interference effects are substantial. Further, our results show that the effects on gait variability are independent from slower walking speeds during DT situations. Deficits in DT performance of children might be explained by the fact that cognitive and muscular capacities of children are most likely restricted by maturational deficits [27]. Krampe et al. [10] were able to show a U-shaped dependency between measures of motor-cognitive performance and age during DT walking. The concurrent performance of a cognitively-demanding task during walking seems to overload children’s cognitive capacities. However, the development of a more unstable gait pattern in children seems to be task-related. Huang et al. [5] demonstrated generally reduced gait velocities during DT walking but the interference effects on gait were largest for an auditory identification task and smallest for a memorization task. This finding indicates that different cognitive tasks affect motor performance in children diversely. The multiple-resource model of attention proposed by Wickens [15] appears to be well-suited to provide an answer to these observations. The model states that 2 tasks will more likely interfere when they share the same pool of cognitive resources. Walking requires central and visual processing; subtracting numbers requires verbal as well as central processing. In addition, subtracting numbers backwards may engage spatial processing when pictured on a time line [28]. In other words, if two tasks are concurrently conducted with the primary task demanding postural control and the secondary task requiring cognitive processing, a decrement in performance of one or both tasks can be observed most likely due to children’s limited cognitive capacity (“central overload”) [29].

Interestingly, previous research mainly focused on cognitive capacities to explain DT decrements. We were able to show a significant relationship between leg muscular capacity and DT walking performance as well. Thus, besides cognitive capacities, leg muscle functions seem to additionally affect DT walking performance in children. Given the association between LTM-LE and leg muscle mass [25], our regression analyses indicate that children with a higher amount of leg muscle mass show shorter step times with lower temporal variability during dual-task walking. These changes are typically attributed to a more unstable gait behavior [30]. A possible explanation for this finding can be derived from learning experiments that demonstrated increased muscle activation in children when executing movements on low performance levels. Improving the quality of the movement (i.e., develop a less variable and more stable performance) reduced the amount of muscle activity and co-contractions needed to coordinate the movement properly [31]. On a neural level, low performance during walking (i.e., large variability) might be accompanied by increased muscle co-contractions. Thus, children with lower lean tissue mass in their lower extremities could be affected by more than one limiting aspect during DT walking. Firstly, they show increased instability during DT walking, which is typically attributed to a cognitive overload [29]. Secondly, their muscular contributions to balance control are insufficient compared to healthy young or middle-aged adults [7]. Given the immature proprioceptive and vestibular sensitivity, more of the child’s attention is required to maintain walking stability, particularly in demanding situations. Furthermore, this more cautious and variable movement is accompanied by an increase in muscle activity [31]. Thus, children with better muscular capacity, especially in their lower extremities, might be able to adequately respond to changes in gait behavior by softening the impact of concurrently ongoing cognitive tasks on their cognitive and motor performance (i.e., freeing up cognitive capacity). As a consequence, they are able to maintain a more stable gait pattern.

## Conclusions

Dual-task situations affect the locomotion of children, irrespectively of their sex. Compared to healthy young and middle-aged adults, children show decreased locomotor performance while walking in cognitive interfering situations. Changes in DT locomotion are typically attributed to limited cognitive information processing. However, we were able to show that besides their cognitive capacities, muscular capacities appear to affect motor performance during DT walking as well. In other words, higher leg lean tissue mass in children may mitigate the impact of a cognitive interference task on DT walking performance by inducing enhanced gait stability.
